# The Effect of Animation Therapy on Time Perception and Daily Routines in Primary School Children: A Randomized Controlled Study

**DOI:** 10.3390/brainsci15111176

**Published:** 2025-10-30

**Authors:** Özgün Belen, Gonca Bumin

**Affiliations:** 1 Tavşanlı Vocational Highschool of Health Services, Kütahya Health Sciences University, Kuütahya 43300, Turkey; 2 Department of Occupational Therapy, Faculty of Health Sciences, Hacettepe University, Ankara 06100, Turkey; gbumin@hacettepe.edu.tr

**Keywords:** time perception, child, animation, executive functions

## Abstract

**Background/Objectives**: Time is a valuable and limited source that must be managed to participate in life domains efficiently. Time perception is an essential skill for managing the time, based on executive functions. This study examined the effects of animation therapy on time perception and daily routines in 7–10-year-old children through executive functions. **Methods**: Sixty-three typically developing children were randomly assigned to intervention and control groups. The intervention group received 10 weeks of animation therapy, while the control group followed daily routines. Time perception was assessed using Duration Discrimination and Reproduction tasks, and daily routines with the Executive Functions and Occupational Routines Scale (EFORTS). **Results**: The intervention group showed significant improvements in Duration Discrimination (500 ms: *p* = 0.02; 800 ms: *p* = 0.01), Duration Reproduction (800 ms: *p* = 0.05), and EFORTS subscales (*p* = 0.00), except Morning and Evening Routines (*p* > 0.05). Medium to large effect sizes were observed for time perception (r = −0.34 to −0.61) and small to large for EFORTS (r = −0.28 to −0.75). The control group showed no to small effects in time perception (r = −0.02 to −0.14) and no to medium effects in EFORTS (r = −0.07 to −0.45). **Conclusions**: Animation therapy may enhance children’s synchronization of time perception with physical time, benefiting social, leisure, and play routines through executive functions. Thus, it could be a valuable addition to occupational therapy interventions.

## 1. Introduction

Time is a limited resource by its nature that we use to accomplish our plans, meet our needs, reach our goals, and hold onto our values. Time perception is a cognitive function related to the subjective experience of the flow and duration of time. The way we perceive time forms a core structure for how we manage this resource, influencing the way we experience life and our success in various areas of life [1,2]. Temporal processing, which constitutes time perception, affects all the behaviors a person exhibits in daily life as it is one of the key components of cognition, behavior, and motor performance and plays an important role in planning future behaviors [3]. Time management is defined in the ICF as “mental functions of ordering events in chronological sequence, allocating amounts of time to events and activities” and largely depends on temporal processing abilities [2,4]. A connection has been shown between time management and work and academic success, job satisfaction, and stress levels [5]. For example, children with Attention Deficit Hyperactivity Disorder (ADHD) who experience difficulties in time management may face problems in carrying out their daily routines. Daily routines, through the efficient use of time, affect occupational performance. Difficulties encountered in situations such as starting and completing a daily activity on time, planning long-term projects, starting and finishing tasks, using a calendar, and rushing when necessary, ultimately affect life and quality of life in areas such as education, self-care, and social life [2].

Our way of perceiving time is subjective, and correspondingly, it is not always synchronized with physical time [6]. Time perception can be influenced by and change due to various internal and external factors [6,7,8]. The nature of both internal and external environmental stimuli, such as duration, complexity, intensity, novelty, and frequency, as well as age, the presence of psychopathologies [6,8], emotional state [6,9], psychiatric medications [9], and the cognitive load required by a task [6], can alter how we perceive time, causing it to speed up or slow down. In a study by Gil et al. with children aged 3–8, it was shown that faces displaying anger were perceived by the children as being shown for longer than they were [10]. A study by Droit-Volet et al. found that children aged 5–8 perceived the durations of auditory and visual sensory stimuli differently [11].

Various models have been developed to explain the mechanisms of time perception that underlie these differences. One such model is the Internal Clock model proposed by Russell M. Church in 1984 [9,10,12,13,14]. According to this model, there is an internal clock composed of three parts: a pacemaker that produces pulses at certain intervals, an accumulator that stores these pulses, and an attention switch that regulates the transmission of pulses from the pacemaker to the accumulator. The pacemaker produces pulses at certain intervals, which begin accumulating in the accumulator when the switch, managed by attention, is closed. Once this accumulation reaches a certain level, the time perception is formed by transmitting the accumulated pulses to the relevant areas of the brain [3,7,10,14]. Events that increase the pacemaker’s pulse production frequency, such as high arousal, cause more pulses to accumulate in a physical unit of time in the accumulator, making the individual perceive time as longer than it actually is. On the other hand, when attention is not focused on the passage of time or when attention is occupied with another task, the attention switch does not transmit all the pulses produced by the pacemaker to the accumulator, resulting in fewer pulses accumulating in the accumulator within a physical unit of time. This leads to the individual perceiving the elapsed time as shorter [3,6,7,14].

Time perception is important in childhood as it is one of the fundamental components of cognition, behavior, and motor skills. It has been shown that impaired time perception in children affects the performance of adaptive behaviors, such as social communication skills and awareness of health and safety [15]. Additionally, the impact of timing functions has been observed in a broad range of performance areas, including verbal skills, expressive music performance, future planning, gross and fine motor skills, and emotional intelligence [16]. Studies have previously shown that time perception training can be conducted in infants as young as 4–5 months [16,17]. Auditory duration discrimination at the millisecond level begins between the ages of 4 and 10 and continues to develop. The period during which the most noticeable change in the ability to detect intervals of silence occurs is identified as between the ages of 7 and 19 [16].

A non-pharmaceutical method that has recently gained attention for its potential to influence time perception and other executive functions is Animation Therapy (AT) [18]. AT is a therapeutic method in which the process of creating stop-motion animation clips is used for therapeutic purposes [19]. These therapeutic purposes can include enhancing cognitive skills such as attention, memory, and self-regulation, improving interpersonal social skills, increasing coping capacities in situations such as pain, grief, and media addiction [20,21], and supporting the rehabilitation of individuals with mental issues; those are socially isolated for various reasons, or those who have been subjected to violence and/or abuse [21,22]. However, there is a limited number of high-level evidence studies in the literature demonstrating the effectiveness of AT in these areas.

AT is based on theories such as the Theory of Development and Sensory Integration Theory and focuses on meeting the physical and emotional needs of the individual [19]. It is a client-centered intervention aiming to maximize their motivation and occupational performance during the therapy process. The source of this motivation comes from the concept of the “just right challenge,” which refers to the perfect balance between the levels of the individual’s skills and the perceived difficulty of the activity [19,23].

AT has an adaptable structure that can be designed according to the individual’s skill level. This allows the difficulty and requirements of the tasks encountered during the therapy process to be aligned with the individual’s skill level. This provides an opportunity for the individual to enter a state of flow [24]. Flow is a positive mental state that arises when the perceived difficulty level of an activity matches the individual’s skill level in a balanced way. It includes enjoyment, a deep state of attention, and a loss of sense of time [23].

Considering all these structural characteristics of AT, the question arises whether it has an impact on cognitive functions such as attention or time perception. There is only one study on the positive effects of AT on attention in primary school children with ADHD [25]. However, there is no literature study on the impact of AT on time perception in children who are typically developing.

In light of all this information, the aim of this study is to investigate whether AT affects time perception and activity routines through executive functions in primary school children.

## 2. Materials and Methods

The study began in May 2023 and ended in November 2024. The reporting of the study followed the Consolidated Standards of Reporting Trials (CONSORT) guidelines [26]. Informed consent forms were obtained from all parents and children who were able to understand the procedure. Ethical approval for the study was granted by a university clinical research ethics committee in February 2023. The study is registered in the clinicaltrials.gov database under ID number NCT05898490.

### 2.1. Study Design

The study was designed as a randomized, controlled, single-blind, 1:1 allocation ratio, two-arm, randomized clinical trial. The required sample size for statistical tests with a power of 80%, Type I error rate of 5%, and effect size of d = 0.34 was determined to be 63 children. Effect size is determined according to the study of Belen in 2019 [25]. Sample size was calculated via “G*Power 3.1” software. Seventy-eight children were found eligible according to the inclusion criteria. Among these, 8 children were excluded due to not meeting the inclusion criteria, and 4 children were excluded for not volunteering to participate, resulting in a total of 12 children being excluded from the study. The study commenced with 66 children who met the inclusion criteria and voluntarily agreed to participate. Each participant was assigned a random study ID that contained no personally identifiable information. An independent researcher outside the study team generated the allocation sequence using a web-based randomization tool (randomizer.org) with 1:1 simple randomization and kept the sequence inaccessible to the investigators involved in screening, enrollment, or assessment. Participant eligibility screening and baseline (pre-intervention) assessments were completed prior to disclosure of group assignment. After each child was enrolled and baseline data were recorded, the independent researcher released the assignment case-by-case to the first author, who then delivered the intervention. Children were allocated to the Intervention Group (IG; n = 33) or Control Group (CG; n = 33).

Group information was accessible only to the first author; the second author remained blinded to allocation and conducted all pre- and post-intervention evaluations for participants in both groups. The IG received animation therapy (AT) delivered by the first author as one-hour sessions, once per week, for 10 weeks. The CG continued their usual activities and education during this period and were placed on a waiting list to receive the intervention after study completion. During the process, 2 children from the IG were excluded due to discontinuity, and 1 child from the CG did not participate in the post-intervention evaluation. Immediately after the 10-week AT period, the second author—still blinded to group assignments—conducted the post-intervention evaluations, and outcome data were collected. Final analysis included 63 children (IG, n = 31; CG, n = 32).

### 2.2. Inclusion and Exclusion Criteria

Children aged 7–10, were typically developing, had no orthopedic, neurodevelopmental, and/or psychiatric diagnoses, and voluntarily agreed to participate in the study were included. During the study process, children who showed discontinuation during the study process, those who did not participate in the pre- and post-intervention evaluations, and those who refused to continue voluntarily were excluded from the study.

### 2.3. Data Collection

A demographic information form was completed to collect data on the children’s age, gender, and grade level. Then, both groups performed the “Duration Discrimination” and “Duration Reproduction” tasks, designed to assess time perception. These assessment tasks have no linguistic or cultural context and provide data in the universal unit of milliseconds. Additionally, the Executive Functions and Occupational Routines Scale (EFORTS) was administered to assess the impact of AT on children’s executive functions. All data collection processes took place in the schools attended by the participating children.

#### 2.3.1. Duration Discrimination Task

As seen in the method used by Toplak in 2005 [27], the time discrimination task is based on the individual’s ability to discriminate the silence duration difference between two successive pairs of stimuli. Typically, the task involves the presentation of two pairs of stimuli, either visually or audibly, of a specific duration of silence interval, and the participant is asked to indicate which pair has the longer or shorter interval of silence [27]. The progression of the test is carried out step-by-step as it follows the Up-Down Transformed-Response Adaptive Procedure (UTRAP) method recommended by Levitt in 1971 [28]. In this method, one of the intervals between the two stimuli is designated as the target duration and the other as the comparison duration. If the individual correctly answers which interval is shorter or longer, the comparison duration is reduced by 15 ms, making the test more difficult. After an incorrect answer, the comparison duration is increased by 15 ms, making the test easier. The test proceeds in this manner until it reaches a total of 6 reversals. The average time differences in the last five reversals are calculated, and the individual’s duration discrimination threshold is determined in milliseconds [28].

**Figure 1:**
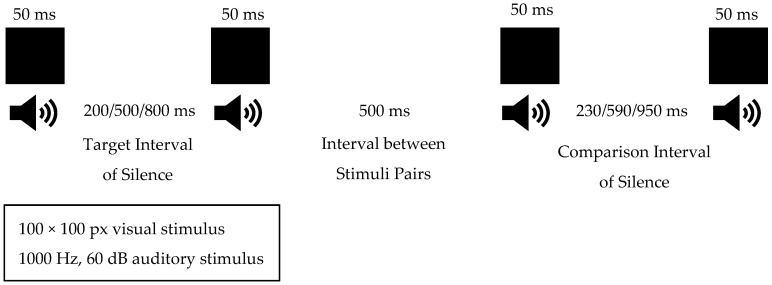
Duration Discrimination Task Flow.

In our study, sensory stimuli that determine interval durations were applied using an assessment method in which both auditory and visual modalities were presented synchronously to isolate the effect of the sensory modality on time perception [11]. A computer software was developed for this method and run via a laptop. When the test was initiated via the software, a black square of 100 × 100 pixels appeared on a white background for 50 ms on the laptop screen, and simultaneously, a 50 ms sine wave tone at 1000 Hz and 60 dB was heard during the 50 ms the square was visible. The duration between the target and comparison stimulus pairs was determined to be 500 ms. In our study, the target interval of silence between stimuli was set to 200 ms, 500 ms, and 800 ms. These durations were chosen based on the information from Huang’s 2012 study, which suggested that shorter durations are more related to the internal clock system and cerebellum, while durations of 800 ms and longer increase the load on working memory [29]. The initial comparison interval of silence duration for 200 ms was set to 230 ms, with a step size of 5 ms. The initial comparison interval of silence duration for the 500 ms was set to 590 ms, with a step size of 15 ms. Finally, the initial comparison interval of silence duration for the 800 ms was set to 950 ms, with a step size of 25 ms (Figure 1). The presentation order of the target and comparison stimuli pairs was random to avoid time-order effects. Additionally, at the beginning of the test, 4 trials were conducted to ensure that the child fully understood the test. In the trials, the target interval of silence duration was 500 ms, and the comparison interval of silence duration was 1000 ms.

#### 2.3.2. Duration Reproduction

**Figure 2:**
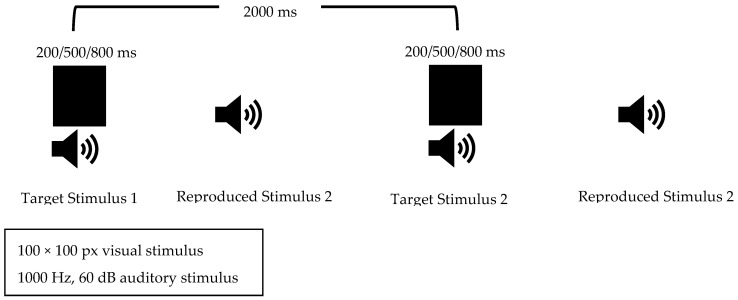
Duration Reproduction Task Flow.

In our study, for the duration reproduction task, the child was asked to produce a stimulus with a duration similar to the stimulus presented, similar to the procedures used in the studies by Wittman et al. (2007) and Yanakieva et al. (2019) [30,31]. A keyboard with a synthesizer was used to allow the child to reproduce the sound. The keyboard’s sound setting was adjusted to match the target stimulus, set to a 1000 Hz sine wave at 60 dB. Visual and auditory stimuli of 200 ms, 500 ms, and 800 ms were used, similar to the stimuli in the duration discrimination task. These stimuli were presented in a completely random order; each was presented 10 times for a total of 30 consecutive stimuli. A 2000 ms interval was left between each stimulus to allow enough time for the child to reproduce the presented stimulus (Figure 2). Before the main part of the test began, one trial was conducted for each duration to ensure the test was understood. The absolute differences between the target durations and each duration the child reproduced were calculated and the ratio of these differences to the target duration was calculated. The average of these ratios was recorded.

#### 2.3.3. Executive Functions and Occupational Routines Scale (EFORTS)

This scale, which evaluates the child’s performance in daily routines, was developed by Frisch and Rosenbaum in 2014 [32]. The scale measures executive functions through the daily routines of children aged 6–12 years from the parent’s perspective. The scale has three subdimensions: morning and evening routines (16 items), play and leisure routines (7 items), and social routines (7 items). Each item is scored on a scale from 1 (never) to 5 (always). Higher scores indicate better performance by the child. The 30 items can be completed in less than 15 min. Confirmatory factor analysis of EFORTS shows good fit measures (CFI = 0.90; RMSEA = 0.06). Also, the internal consistency of the scale was determined as high (0.83–0.92), and internal reliability was α = 0.947 [32]. The Turkish adaptation of the scale has been reported to have excellent test–retest reliability with an ICC of 0.91 [33].

### 2.4. Intervention

The characteristics of the intervention program, including its content, duration, and frequency, were set considering previous studies on AT [25,34,35]. The evaluation and AT sessions were scheduled according to the availability of the children’s class schedules. The sessions took place in a quiet office at the children’s schools, with a laptop, a camera, and necessary props for filming (such as decorations and other items used during the film shoot) on a desk. The room’s curtains were closed, and sessions were conducted when all other children were in their classes for lesson time, ensuring the school was quiet to avoid visual and auditory distractions. AT intervention’s content cannot be standardized since it is a client-adaptive, individualizable occupational therapy intervention. However, the 10-week AT intervention process was generally planned as follows:

- Session 1—Introduction to animation and the AT process, viewing sample animations demonstrating filming techniques;
- Session 2—Practical application of the learned techniques;
- Sessions 3 and 4—Determining the movie’s topic and filming techniques, preparing the movie’s script, followed by the creation of necessary sets, scenes, and characters as per the script;
- Sessions 5 to 8—Creating the frames and filming process;
- Session 9—Voice-over of the film, combining the visuals and sound recordings with necessary adjustments;
- Session 10—Watching the completed film with the family; receiving comments and feedback from the family regarding the film; the child expressing their feelings and thoughts about the film and the filming process; delivering the final film to the child and family.

This protocol was applied for 10 weeks as one session per week for a total of ten sessions. Session duration varied between 30–60 min; the total duration of 10 sessions is 430 min. The details of the AT protocol, session by session, will be provided as a Supplementary File S1.

### 2.5. Statistics

The data obtained from the two groups in the study were evaluated through visual and numerical analyses based on their compliance with normal distribution. Histogram, Q-Q plot, Kolmogorov–Smirnov test, skewness, and kurtosis values were examined for these analyses, revealing that the data did not follow a normal distribution. Therefore, the Wilcoxon matched-pairs signed-rank test was used for within-group comparisons, and the Mann–Whitney U test was used for between-group comparisons. Pearson’s r was used to calculate the effect size of the groups revealed. When interpreting Pearson’s r values, the limit values are determined as $\left|r\right|$ between 0.1–0.3: small, 0.3–0.5: medium, and higher than 0.5: large [36]. Statistical analyses were performed using IBM SPSS Statistics, version 23.0 (IBM Corp., Armonk, NY, USA)

## 3. Results

**Figure 3:**
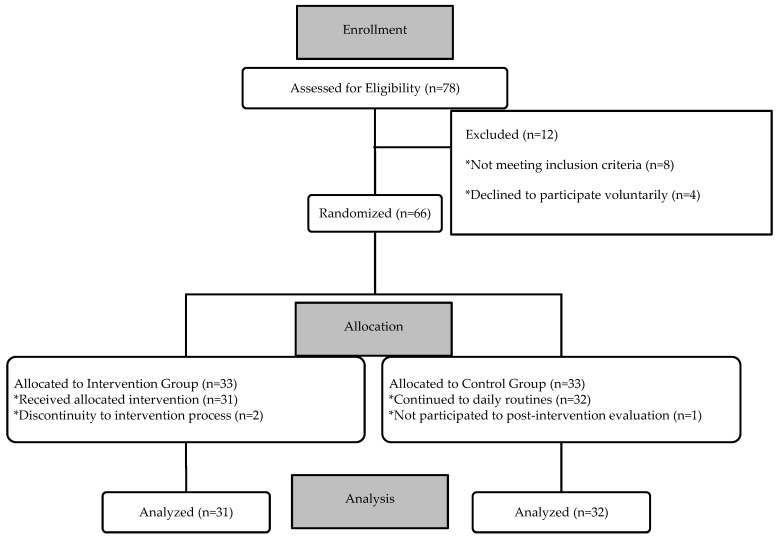
CONSORT Flowchart of the Participants.

The study initially commenced with 78 children. Twelve were excluded before participation; 8 did not meet the inclusion criteria, and 4 declined to participate, leaving 66 consented participants. During the intervention process, 2 children in the intervention group discontinued, and 1 child in the control group did not complete the post-intervention assessment. Thus, the final analysis included data from 63 children (Figure 3).

### 3.1. Demographics and Pre-Intervention Evaluations of the Groups

No statistically significant difference was found between IG and CG in age (*p* = 0.54) and gender (*p* = 0.49). Both groups’ pre-intervention scores for all outcomes were found to be similar (Table 1).

### 3.2. Comparison of Pre- and Post-Intervention Values Within Groups

In the IG, statistically significant differences were found for all values except for EFORTS—Morning and Evening Routines (*p* = 0.13) and Duration Reproduction 200 ms (*p* = 0.07). All other scores showed a positive change for the perception of time, which became more synchronized with physical time (*p* < 0.05) (Table 2).

In the CG, significant differences were observed for EFORTS—Morning and Evening Routines (*p* = 0.01) and the EFORTS total score (*p* = 0.04), while no statistically significant differences were found in other scores (*p* > 0.05) (Table 2).

When examining effect sizes, all Pearson’s r values of the IG ranged from medium to large (r = −0.28 to −0.75), but EFORTS—Morning and Evening Routines had a small effect size (r = −0.28). Effect sizes of CG ranged from no to medium (r = −0.02 to −0.45) (Table 2).

### 3.3. Comparison of Pre- and Post-Intervention Differences in the Study and Control Groups

When comparing the differences between pre- and post-intervention evaluations for the two groups, a significant difference was revealed between the two groups for all other measurements (*p* < 0.05) except Duration Discrimination 200 ms (*p* = 0.06), Duration Reproduction 200 ms (*p* = 0.28), Duration Reproduction 500 ms (*p* = 0.16), and EFORTS—Morning and Evening Routines (*p* = 0.33) (Table 3).

## 4. Discussion

The aim of this study was to examine the effects of AT on time perception and occupational routines through executive functions in primary school children. It was found that AT had a potential effect on synchronizing the time perception of primary school children with physical time. It was also demonstrated that this revealed effect had positive impacts on children’s play, leisure, and social routines through executive functions. Also, it should be noted that this study is the only clinical trial study that investigates the effects of animation therapy on typically developing children, so that the discussion will be compulsorily conducted with studies on certain diagnostic groups.

At the end of the 10-week AT intervention in the IG, it was observed that time perception became more synchronized with physical time in all categories of the duration discrimination tasks and in the duration reproduction tasks at 500 ms and 800 ms durations. When examining the effect size of these revealed changes, it was found that the change in the IG was larger than in the CG. Previous studies have shown that time perception is influenced by factors such as mood, arousal, and attention. For example, in a study by Droit-Volet, a duration discrimination test consisting of three difficulty levels was performed on children aged 5 to 8 years. In the test, children’s mood was examined by using images of faces with neutral and angry expressions, and their effect on time perception was investigated. As a result of the study, it was determined that angry face images created a perception of time passing more slowly than it actually was [37]. Similarly, in a study by Nazari et al., images were shown in pairs at different durations to children with and without ADHD to create positive, negative, and neutral emotions. Children were asked which image was shown for a longer or shorter period, and the duration discrimination thresholds were determined. The study found that, regardless of ADHD status, both groups showed significant differences in the duration discrimination thresholds for positive and negative images compared to neutral images [38]. In a study by Zakay in 1992 with children aged 7 to 9 years, another factor affecting time perception was shown to be attention skills [39]. In a 2020 meta-analysis study, Nejati et al. concluded that ADHD affects children’s time perception, especially in the estimation of longer durations [40]. Another important study by O’Connell showed the effect of arousal regulation on attention skills. The study found that training aimed at voluntarily regulating arousal improved sustained attention skills [41]. All these studies are consistent with the results observed in this study. The skill level required for the AT process can be adjusted according to the individual’s skill level [24]. When the perceived difficulty level matches the individual’s skill level, it helps regulate the arousal level to the optimal point and induces a flow state [42]. The regulation of arousal and improvement of attention skills through AT have also been shown in Belen’s 2019 study [25]. Considering this information, we can interpret that the AT applied to the IG regulated the arousal level to the optimal point, leading to a positive effect on attention skills and, consequently, synchronizing time perception with physical time.

In the duration reproduction tasks specified in the time perception measurement methods, AT revealed a significant effect in the 500 ms and 800 ms duration reproduction tasks, while no effect was observed in the 200 ms duration reproduction values of the IG. The duration reproduction task is the task with the highest workload among the time estimation tasks. In addition to the attention workload required for temporal processing, the reproduction of time requires the stimuli presented during the task to be held in working memory throughout the motor performance [43]. In a study by Wallace, it was stated that the shortening of the required duration increased the workload revealed in the time perception reproduction process [44]. In a study by Koch, it was stated that the cerebellum is one of the essential components in processing short durations [45]. In relation to this information, when examining the development of the cerebellum according to age, a study by Tiemeier showed that cerebellar development peaks at 11.8 years in girls and 15.6 years in boys [46]. In a study by Wierenga, it was shown that cerebellar development reaches its peak at 15.6 years in boys, consistent with Tiemeier’s study [47]. In light of this information, the fact that AT did not reveal a significant effect in the 200 ms duration reproduction measurements and that the effect size was relatively smaller raises the question that both the complexity of the duration reproduction task and the difficulty of reproducing such a short 200 ms duration could be a challenging task for motor skills for the age range in our study.

When examining the EFORTS—Play and Leisure Routines, EFORTS—Social Routine, and EFORTS total scores, the statistically significant differences and effect sizes revealed after the intervention in the IG showed that AT positively affected the performance of daily routines through its impact on executive functions. Managing daily routines requires the effectiveness of many executive functions, including temporal processing, which is involved and interacts with them. Frisch et al. suggested that delays or reductions in the development of executive functions could be an indicator of social and behavioral problems in children [32]. Children with conditions affecting executive functions, such as ADHD, Autism, and Learning Disabilities, are noted to experience undeniable difficulties in independently carrying out their daily routines [32]. Additionally, evidence from Belen’s 2019 study showed that AT improved executive functions, such as attention and behavioral inhibition, in children with ADHD [25]. We believe that the improvements observed in social, play, and leisure routines in this study were revealed through the positive effects of AT on executive functions like time perception, attention, and behavioral inhibition, which underpin these routines.

AT showed no effect on morning/evening routines or on 200-ms tasks, yet produced clear gains at longer time intervals. We interpret this dissociation in light of cognitive load. Discriminating very brief sub-second durations (200 ms) is dominated by low-level temporal resolution with minimal reliance on executive resources, whereas maintaining and comparing longer temporal representations places greater demands on working memory—the putative target of AT intervention. Consistent with this view, recent evidence on the EFORTS indicates that items most relevant to morning and evening routines cluster with working-memory processes [32]. Yet, translating cognitive improvements into routinized daily behaviors may additionally require environmental scaffolding (e.g., structured cues at home/school), habit formation, and time for generalization, which may explain the limited change observed in those activities over the 10-week program.

Participation in creative processes provides opportunities for children to develop self-esteem, autonomy, and competence. Art therapy creates safe spaces and opportunities for these creative processes and influences the child’s ability to express their feelings, thoughts, and needs [48]. AT inherently involves creative processes like art therapy and has a structure that can create opportunities for children to express themselves. Throughout the process, the child experiences expressing their inner world and emotions through an animated film. Including the child in the animation film production and the active role of the therapist in the filming process provide the child with the opportunity for social interaction [24]. In line with Blount’s study, we believe that as a result of the increased capacity to recognize and express emotions gained through AT, children become more integrated into social routines [49]. The development observed in EFORTS—Play and Leisure Routines and Social Routine can be seen as arising through this mechanism revealed by AT.

### Limitations and Future Implications

In this study, the effect of AT on time perception was examined in terms of synchronizing it with physical time. Future studies could determine whether AT has an effect on slowing down or speeding up the perceived time. Also, this study focused on sub-second intervals (200–800 ms) to target fundamental perceptual timing mechanisms implicated in attention/arousal coupling in school-age children; future work will extend to second–minute ranges and subjective time judgments to enhance ecological validity. The effect of AT on executive functions on daily routines was measured using EFORTS. EFORTS is a parent-reported scale that does not evaluate the executive functions of children directly, possibly leading to subjective bias. Future studies could use different measurement tools like Stroop, the Trail Making test or TEA-Ch that assess the performance of the child directly. Also, including the assessment methods like EEG or fMRI in future studies may provide further information on neurophysiological mechanisms underlying time perception and the effects of the interventions that were investigated. Since this study was conducted on children aged 7–10, time perception evaluations were performed using methods that were as simple as possible. Studies conducted on adults could include more complex methods, such as tests involving verbal components. As cognitive skills develop in adults, the scope of time perception also evolves. Investigating how longer durations (seconds, minutes, hours, days) are affected by AT in adults would provide new insights into time perception skills in literature. This would allow for new information related to the neurophysiological and neuroanatomical structure of the internal clock to be gathered. Also, this study used an AT protocol which lasts 10 weeks and did not administer a follow-up assessment to evaluate the durability of the effects of AT. Future studies may explore the long-term effects of an AT intervention that lasts longer. Because the present study was conducted in schools serving students residing within a limited geographic area, its external validity may be constrained. Future research should employ samples that ensure greater cultural diversity. This study compared the effects of AT only with a waiting list. Future studies may include another intervention group similar to AT to rule out the placebo effect. Last but not least, this study was conducted with typically developing children only. Future studies on the interaction of AT with groups that have performance problems with time perception (neurodivergent or clinical groups like ADHD) may help enhance the body of knowledge on time perception.

## 5. Conclusions

This study examined the effects of a 10-week AT intervention on time perception and daily routines related to executive functions in typically developing primary school children. It was found that AT affected elements such as arousal and attention, which are believed to contribute to the internal clock that forms time perception, synchronizing it more closely with physical time. The changes in time perception revealed at the end of the AT process were reflected in the execution of daily routines related to sociality and leisure. We can conclude that AT would be a valuable addition to occupational intervention programs.

### 5.1. Key Findings

- The 10-week AT intervention has significant potential to improve children’s time perception, making their internal clocks more synchronized with physical time;
- AT may positively influence the children’s performance in managing daily routines such as play, leisure, and social activities by affecting their executive functions.

### 5.2. What This Study Added

This study revealed that AT has the potential to enhance time perception in typically developing children, synchronizing their internal clock with physical time. It also shows that AT may positively affect daily routine performance, such as with social, play, and leisure activities. All these suggest that AT has the potential to be an effective intervention in occupational therapy for improving children’s daily occupational performance.

## Figures and Tables

**Table 1:** Comparison of the Groups’ Demographics and Pre-intervention Scores.

		IG		CG		Z	*p*
		M (Years)	SD	M (Years)	SD		
Age		8.32	1.33	8.47	1.16	−0.61	0.54
		N		N			
Gender	Male	21		19		−0.68	0.49
	Female	10		13			
		M (ms)	SD	M (ms)	SD		
DDT	200 ms	28.29	12.24	28.88	11.71	−0.28	0.78
	500 ms	60.77	15.97	62.34	16.96	−0.19	0.85
	800 ms	116.29	52.87	117.34	53.58	−0.03	0.98
DRT	200 ms	39.05	18.46	42.28	19.42	−0.51	0.61
	500 ms	30.92	16.52	33.03	18.23	−0.37	0.71
	800 ms	17.80	4.98	18.48	5.03	−0.52	0.60
		M	SD	M	SD		
EFORTS	MaER	3.64	0.65	3.64	0.65	−0.01	0.99
	PaLR	4.17	0.60	4.24	0.58	−0.48	0.63
	SR	3.67	0.62	3.71	0.61	−0.39	0.70
	TS	3.83	0.58	3.86	0.44	−0.10	0.92

IG: Intervention Group; CG: Control Group; Z: Z-score; *p*: probability value; M: Mean; SD: Standard Deviation; N: number of participants; ms: milliseconds; DDT: Duration Discrimination Task; DRT: Duration Reproduction Task; EFORTS: Executive Functions and Occupational Routines Scale; MaER: Morning and Evening Routines; PaLR: Play and Leisure Routines; SR: Social Routines; TS: Total Score.

**Table 2:** Effect Sizes and Comparison of Pre- and Post-Intervention Values Within Groups.

		IG			CG		
		Z	Asymp. Sig. (2-Tailed)	r	Z	Asymp. Sig. (2-Tailed)	r
DDT	200 d	−2.15	0.03 *	−0.39 ^b^	−0.10	0.92	−0.02
	500 d	−2.59	0.01 *	−0.47 ^b^	−0.69	0.49	−0.13 ^a^
	800 d	−3.36	0.01 *	−0.61 ^c^	−0.71	0.48	−0.13 ^a^
DRT	200 d	−1.94	0.07	−0.34 ^b^	−0.43	0.67	−0.08
	500 d	−2.18	0.03 *	−0.40 ^b^	−0.74	0.46	−0.14 ^a^
	800 d	−2.10	0.04 *	−0.38 ^b^	−0.31	0.76	−0.06
EFORTS	MaERd	−1.54	0.13	−0.28 ^a^	−2.51	0.01 *	−0.45 ^b^
	PaLRd	−3.80	0.00 *	−0.69 ^c^	−0.36	0.72	−0.07
	SRd	−4.13	0.00 *	−0.75 ^c^	−0.68	0.50	−0.12 ^a^
	TSd	−3.65	0.00 *	−0.66 ^c^	−2.03	0.04 *	−0.36 ^b^

* *p* < 0.05; ^a^ −0.1> r > −0.3; small effect size; ^b^ −0.3 > r > −0.5; medium effect size; ^c^ −0.5 > r > −1.0; large effect size; IG: Intervention Group; CG: Control Group; Z: Z-score; Asymp. Sig.: Asymptotic Significance; r: Pearson’s r value; DDT: Duration Discrimination Task; DRT: Duration Reproduction Task; EFORTS: Executive Functions and Occupational Routines Scale; 200 d: difference in 200 ms tasks; 500 d: difference in 500 ms tasks; 800 d: difference in 800 ms tasks; MaERd: Morning and Evening Routines score difference; PaLRd: Play and Leisure Routines score difference; SRd: Social Routines score difference; TSd: Total Score difference.

**Table 3:** Comparison of Pre- and Post-Intervention Differences between Groups.

		IG		CG			
		M (ms)	SD	M (ms)	SD	Z	Asymp. Sig. (2-Tailed)
DDT	200 d	4.03	10.13	0.09	5.61	−1.89	0.06
	500 d	8.39	19.32	2.22	11.42	−2.36	0.02 *
	800 d	27.10	37.70	3.75	24.66	−2.54	0.01 *
DRT	200 d	4.18	17.23	2.51	13.04	−1.07	0.28
	500 d	3.50	14.13	0.17	6.19	−1.42	0.16
	800 d	2.23	6.05	0.06	2.75	−1.98	0.05 *
		M	SD	M	SD		
EFORTS	MaERd	−0.18	0.51	−0.10	0.19	−0.97	0.33
	PaLRd	−0.30	0.32	−0.02	0.17	−3.83	0.00 *
	SRd	−0.44	0.47	−0.01	0.11	−3.63	0.00 *
	TSd	−0.28	0.34	−0.04	0.10	−3.24	0.00 *

* *p* < 0.05; IG: Intervention Group; CG: Control Group; M: Mean; ms: milliseconds; SD: Standard Deviation; Z: Z-score; Asymp. Sig.: Asymptotic Significance; DDT: Duration Discrimination Task; DRT: Duration Reproduction Task; EFORTS: Executive Functions and Occupational Routines Scale; 200 d: difference in 200 ms tasks; 500 d: difference in 500 ms tasks; 800 d: difference in 800 ms tasks; MaERd: Morning and Evening Routines score difference; PaLRd: Play and Leisure Routines score difference; SRd: Social Routines score difference; TSd: Total Score difference.

## Data Availability

The data that support the findings of this study are available on request from the corresponding author. The data are not publicly available due to privacy/ethical restrictions (vulnerable group: child).

## Supplementary Materials

The following supporting information can be downloaded at: https://www.mdpi.com/article/10.3390/brainsci15111176/s1, File S1: Weekly Animation Therapy Intervention Program.

## Abbreviations

The following abbreviations are used in this manuscript:

AT	Animation Therapy
EFORTS	Executive Functions and Occupational Routines Scale
ADHD	Attention Deficit Hyperactivity Disorder

## References

[1] Siu N.Y., Lam H.H., Le J.J., Przepiorka A.M. (2014). Time perception and time perspective differences between adolescents and adults. Acta Psychol..

[2] Wennberg B., Janeslätt G., Gustafsson P.A., Kjellberg A. (2021). Occupational performance goals and outcomes of time-related interventions for children with ADHD. Scand. J. Occup. Ther..

[3] Coelho M., Ferreira J.J., Dias B., Sampaio C., Martins I.P., Castro-Caldas A. (2004). Assessment of time perception: The effect of aging. J. Int. Neuropsychol. Soc..

[4] WHO Organization (2001). International Classification of Functioning, Disability, and Health: ICF.

[5] Mette C. (2023). Time perception in adult ADHD: Findings from a Decade—A Review. Int. J. Environ. Res. Public Health.

[6] Appelqvist-Dalton M., Wilmott J.P., He M., Simmons A.M. (2022). Time perception in film is modulated by sensory modality and arousal. Atten. Percept. Psychophys..

[7] Behm D.G., Carter T.B. (2020). Effect of exercise-related factors on the perception of time. Front. Physiol..

[8] Fenner B., Cooper N., Romei V., Hughes G. (2020). Individual differences in sensory integration predict differences in time perception and individual levels of schizotypy. Conscious. Cogn..

[9] Droit-Volet S., Meck W.H. (2007). How emotions colour our perception of time. Trends Cogn. Sci..

[10] Gil S., Niedenthal P.M., Droit-Volet S. (2007). Anger and time perception in children. Emotion.

[11] Droit-Volet S., Meck W.H., Penney T.B. (2007). Sensory modality and time perception in children and adults. Behav. Process..

[12] Church R.M. (1984). Properties of the internal clock. Ann. N. Y. Acad. Sci..

[13] Gibbon J., Church R.M., Meck W.H. (1984). Scalar timing in memory. Ann. N. Y. Acad. Sci..

[14] Kramer R.S., Weger U.W., Sharma D. (2013). The effect of mindfulness meditation on time perception. Conscious. Cogn..

[15] Meaux J.B., Chelonis J.J. (2003). Time perception differences in children with and without ADHD. J. Pediatr. Health Care.

[16] Noreika V., Falter C.M., Rubia K. (2013). Timing deficits in attention-deficit/hyperactivity disorder (ADHD): Evidence from neurocognitive and neuroimaging studies. Neuropsychologia.

[17] Droit-Volet S. (2013). Time perception in children: A neurodevelopmental approach. Neuropsychologia.

[18] Choo H. (2015). A New Approach to Art Therapy Using Animation: Animation Therapy: Animation Therapy. TECHART J. Arts Imaging Sci..

[19] Mason H. (2011). The re-animation approach: Animation and therapy. J. Assist. Technol..

[20] Park S.-H., Kim S.-Y., Choo J.H.-J., Lee W.J., Kang J.-S. Using new media to create integrating art therapy: Animation therapy. Proceedings of the ACM SIGGRAPH ASIA 2009 Educators Program 2009.

[21] Tabana D. (2024). Animation as Therapy for Mental Health Treatment Across Diverse Populations and Contexts, A Literature Review. Ph.D. Thesis.

[22] Hani M. (2017). Defining animation therapy: The good hearts model. Animat. Pract. Process Prod..

[23] Csikszentmihalyi M. (2013). Flow: The Psychology of Happiness.

[24] Mason H.R. (2009). Dare to Dream: The use of animation in occupational therapy. Ment. Health Occup. Ther..

[25] Belen Ö. (2019). Animasyon Terapisinin Dikkat Eksikliği ve Hiperaktivite Bozukluğu olan Çocuklarda Dikkat ve Dürtüsellik Seviyelerine Etkisi. Master’s Thesis.

[26] Schulz K.F., Altman D.G., Moher D. (2010). *CONSORT* 2010 statement: Updated guidelines for reporting parallel group randomised trials. J. Pharmacol. Pharmacother..

[27] Toplak M.E., Tannock R. (2005). Time perception: Modality and duration effects in attention-deficit/hyperactivity disorder (ADHD). J. Abnorm. Child Psychol..

[28] Levitt H. (1971). Transformed up-down methods in psychoacoustics. J. Acoust. Soc. Am..

[29] Huang J., Yang B.-R., Zou X.-B., Jing J., Pen G., McAlonan G.M., Chan R.C.Y. (2012). Temporal processing impairment in children with attention-deficit-hyperactivity disorder. Res. Dev. Disabil..

[30] Wittmann M., Carter O., Hasler F., Cahn B.R., Grimberg U., Spring P., Hell D., Flohr H., Vollenweider F.X. (2007). Effects of psilocybin on time perception and temporal control of behaviour in humans. J. Psychopharmacol..

[31] Yanakieva S., Polychroni N., Family N., Williams L.T., Luke D.P., Terhune D.B. (2019). The effects of microdose LSD on time perception: A randomised, double-blind, placebo-controlled trial. Psychopharmacology.

[32] Frisch C., Rosenblum S. (2014). Reliability and validity of the executive function and occupational routines scale (EFORTS). Res. Dev. Disabil..

[33] Akyürek G., Bumin G. (2017). Turkish Adaptation of The Executive Functions and Occupational Routines Scale (EFORTS) and Its Validity and Reliability. Dev. Med. Child Neurol..

[34] Bailes A.F., Reder R., Burch C. (2008). Development of guidelines for determining frequency of therapy services in a pediatric medical setting. Pediatr. Phys. Ther..

[35] Oyanadel C., Buela-Casal G., Araya T., Olivares C., Vega H. (2014). Percepción del tiempo: Resultados de una intervención grupal breve para el cambio del perfil temporal. Suma Psicológica.

[36] Cohen J. (2013). Statistical Power Analysis for the Behavioral Sciences.

[37] Droit-Volet S., Fayolle S., Gil S. (2016). Emotion and time perception in children and adults: The effect of task difficulty. Timing Time Percept..

[38] Nazari M.A., Mirloo M.M., Rezaei M., Soltanlou M. (2018). Emotional stimuli facilitate time perception in children with attention-deficit/hyperactivity disorder. J. Neuropsychol..

[39] Zakay D. (1992). The role of attention in children’s time perception. J. Exp. Child Psychol..

[40] Nejati V., Yazdani S. (2020). Time perception in children with attention deficit–hyperactivity disorder (ADHD): Does task matter? A meta-analysis study. Child Neuropsychol..

[41] O’Connell R.G., Bellgrove M.A., Dockree P.M., Lau A., Fitzgerald M., Robertson I.H. (2008). Self-Alert Training: Volitional modulation of autonomic arousal improves sustained attention. Neuropsychologia.

[42] Czikszentmihalyi M. (1990). Flow: The Psychology of Optimal Experience.

[43] Barkley R.A., Edwards G., Laneri M., Fletcher K., Metevia L. (2001). Executive Functioning, Temporal Discounting, and Sense of Time in Adolescents with Attention Deficit Hyperactivity Disorder (ADHD) and Oppositional Defiant Disorder (ODD). J. Abnorm. Child Psychol..

[44] Wallace G.L., Happé F. (2008). Time perception in autism spectrum disorders. Res. Autism Spectr. Disord..

[45] Koch G., Oliveri M., Torriero S., Salerno S., Gerfo E.L., Caltagirone C. (2007). Repetitive TMS of cerebellum interferes with millisecond time processing. Exp. Brain Res..

[46] Tiemeier H., Lenroot R.K., Greenstein D.K., Tran L., Pierson R., Giedd J.N. (2010). Cerebellum development during childhood and adolescence: A longitudinal morphometric MRI study. NeuroImage.

[47] Wierenga L., Langen M., Ambrosino S., van Dijk S., Oranje B., Durston S. (2014). Typical development of basal ganglia, hippocampus, amygdala and cerebellum from age 7 to 24. NeuroImage.

[48] Councill T. (2003). Medical art therapy with children. Handb. Art Ther..

[49] Blount B.G. (1984). The language of emotions: An ontogenetic perspective. Lang. Sci..

