# Supplementary material for: The Effect of Animation Therapy on Time Perception and Daily Routines in Primary School Children: A Randomized Controlled Study
Source: Brain Sci. 2025 Oct 30;15(11):1176. doi: 10.3390/brainsci15111176 (PMC12650145; doi:10.3390/brainsci15111176)
Supplement: Supplementary file 1 [file brainsci-15-01176-s001.zip › brainsci-3925851-supplementary.pdf]

Supplementery Material File S1. Weekly Animation Therapy Intervention Program

Week	Durati on	Activity	Description	Goals
1st	45 min.	**Introduction of Stop Motion Animation**	Animation therapist introduces stop motion animation and its basic principles to child using devices like zoetrope and flipbooks. The therapist and the child together watches stop motion animation clips produced with five different techniques; draw animation, claymation, pixilation, object animation, and cut-out animation.	To create and/or raise awareness about stop motion animation in children. To demonstrate the "frames to animation" concept (how the still frames becomes alive) and the process of stop motion animation production to the child. To explore the effects of technique used in the stop motion animation, how they differ from each other.
2nd	60 min.	**Practicing the Techniques**	The child and the therapist produce a total of five video clips, using each five technique seperately. Clips' durations were 4-5 seconds long (48-60 frames), just enough to experience each technique's dynamics, principles, and feeling.	To gain deeper knowledge about stop motion animation techniques enough to be able to decide which technique the child will use in his/her animation.
3rd	45 min.	**Creating the Script**	The child creates a script and the therapist provides support as the child request. The extent and complexity may vary due to age of the children. Scenarios created by younger children may be "a doughball rolling around" or "a toy car passes by a pile of paper clips". Older children may create more complex scenarios like "two doughballs having a conversation about how hard was their last math exam" or "a toy car race with close-calls and comebacks". Therapist writes down the script for recording.	To create a script for the child's actual stop motion animation. This script guides the preparation of the props and scenery and also the shooting process.
4th	55 min.	**Production of Props and Scenery**	The child and the terapist produces the props and sceneries as they planned. This process shows variations due to the content of the script the child created. For example; if cut-out animation is preferred, the child and therapist selects suitable pictures for their characters and scenery and cut them out from magazines. For another example; if the child preferred object animation, the child decides what objects will be used as characters, props and scenery in his/her animation.	To produce necessary items (drawing of the characters, objects, scene backgrounds, etc.) for production of the child's stop motion animation.

Week	Duration	Activity	Description	Goals
**5th-8th**	**60 min.**	**Shooting the Frames**	The child animates the characters and/or props due to his/her script as "the animator". The therapist shoots the frame using stop motion animation software installed on the computer as "the cameraman". They may change the roles time to time if the child wants to. At the end of the 8th session, all the frames created by the child and the therapist are brought together and become an animation video (an .mp4 file) through stop motion animation software.	In general, stop motion animations uses the framerate of 15/sec. So the goal of one session of shooting is to produce at least 10-15 sec (150-225 frames) clip. At the end of the 8th session, the aim is for the clips to be at least 60-90 sec long. Duration of the animation may be longer due to the script the child created.
**9th**	**45 min.**	**-Dubbing, Determining and Editing Sound Effects and Background Musics.**<br>**-Finalizing and Exporting Video Clip**	-The child and therapist makes voice acting together to dub the characters (if any); the child chooses the sound effects and background musics. Child decides how to use sound effects and background music and therapist uses the software to do the editing as the child lead.<br>-The therapist exports the finalized animation video clip via software. They together watch the final version of the stop moiton animation clip. They make the necessary corrections if needed.	-To produce the sound tracks of the stop motion animation.<br>-To obtain the final version of the animation clip and finalize the production process of stop motion animation.
**10th**	**30 min.**	**-Ending Sessions**<br>**-Taking Feedbacks**	-The only copy of the animation clip's .mp4 file is delivered to family via a digital medium (flash drive, e-mail, etc.). Therapist does not keep any copy of the animation clip or any file since it is irrelevant to therapy process. Lastly good wishes are exchanged and farewells are said.<br>-The Family makes their comments about animation movie and congratulate their child for his/her efforts. The child, the family and the therapist have a conversation about the whole process and sessions.	-To deliver the final product to family and conclude the animation therapy process.<br>-To create a positive atmosphere where the final product's quality is not the main consideration but the efforts made by child, his/her experiences and feelings during the sessions.

Materials Needed:

- Flipbook, Zoetrope
- Camera
- Computer (with a stop motion animation software installed)
- Blank papers, pen, coloring pencils, crayons, scissors, eraser, tack-it, old magazines (for Drawing animation and Cut-out techniques)
- Play doughs (for Claymation technique)
- Variety of objects (like beads, toys, fruits, paper clips etc., for Object Animation technique)
- Flash drive (or any medium similiar for delivering the animation .mp4 file)
